# Oral drug delivery for immunoengineering

**DOI:** 10.1002/btm2.10243

**Published:** 2021-08-10

**Authors:** Tien Le, Brian Aguilar, Joslyn L. Mangal, Abhinav P. Acharya

**Affiliations:** ^1^ Chemical Engineering, School for the Engineering of Matter, Transport, and Energy Arizona State University Tempe Arizona USA; ^2^ Biomedical Engineering, School of Biological and Health Systems Engineering Arizona State University Tempe Arizona USA; ^3^ Biological Design, School for Biological and Health Systems Engineering Arizona State University Tempe Arizona USA; ^4^ Materials Science and Engineering, School for the Engineering of Matter, Transport, and energy Arizona State University Tempe Arizona USA; ^5^ Biodesign Center for Immunotherapy, Vaccines and Virotherapy Arizona State University Tempe Arizona USA

**Keywords:** immunotherapy, oral bioavailability, oral immunotherapeutic delivery, oral vaccination

## Abstract

The systemic pharmacotherapeutic efficacy of immunomodulatory drugs is heavily influenced by its route of administration. A few common routes for the systemic delivery of immunotherapeutics are intravenous, intraperitoneal, and intramuscular injections. However, the development of novel biomaterials, in adjunct to current progress in immunoengineering, is providing an exciting area of interest for oral drug delivery for systemic targeting. Oral immunotherapeutic delivery is a highly preferred route of administration due to its ease of administration, higher patient compliance, and increased ability to generate specialized immune responses. However, the harsh environment and slow systemic absorption, due to various biological barriers, reduces the immunotherapeutic bioavailability, and in turn prevents widespread use of oral delivery. Nonetheless, cutting edge biomaterials are being synthesized to combat these biological barriers within the gastrointestinal (GI) tract for the enhancement of drug bioavailability and targeting the immune system. For example, advancements in biomaterials and synthesized drug agents have provided distinctive methods to promote localized drug absorption for the modulation of local or systemic immune responses. Additionally, novel breakthroughs in the immunoengineering field show promise in the development of vaccine delivery systems for disease prevention as well as combating autoimmune diseases, inflammatory diseases, and cancer. This review will discuss current progress made within the field of biomaterials and drug delivery systems to enhance oral immunotherapeutic availability, and how these new delivery platforms can be utilized to deliver immunotherapeutics for resolution of immune‐related diseases.

## 1 INTRODUCTION

Drug delivery is the process of administering a pharmaceutical compound in order to achieve a therapeutic effect. Oral administration is a preferred method for delivery because of its convenient and noninvasive delivery of drugs. However, a variety of obstacles limit the efficacy of oral drug administration, namely, the acidic and enzymatic degradation in the stomach, the range of pH throughout the gastrointestinal (GI) tract (pH ranging from 1 to 7), first‐pass metabolism, the steric barrier of the mucosal system and the physical barrier of the epithelial layers, to name a few. Each of these challenges contribute to the complex course an immunotherapeutic has to take through the body's intricate GI tract prior to reaching its target location within the intestine for absorption and systemic bioavailability. For the purpose of this review, bioavailability is defined as the potential of orally delivered drugs to reach systemic circulation. These issues are further escalated and complicate delivery of biologics such as vaccines and antibodies that need to be delivered systemically for modulating disease outcomes. Biomaterials and drug delivery systems (DDS) can play an important role in developing strategies to overcome these issues and allow for delivery of therapeutics to the immune system locally in mucosa or systemically. Notably for delivery of fragile immunotherapeutics, such as antibodies, mRNA, and DNA, specialized DDS are required, which can overcome the challenges associated with oral delivery. While there have been recent reviews that also suggest the importance of DDS for oral delivery, ^1^, ^2^, ^3^, ^4^ this review will focus on discussion of immune engineering and immunotherapeutic delivery via oral route. In this review, we will first discuss the major immunotherapeutics that can potentially be delivered orally, and challenges associated with poor oral drug targeting. Next, we will discuss current oral to systemic delivery strategies, specific delivery mechanisms, and the promising future of oral drug administration for the systemic modulation of immune responses.

## 2 IMMUNOTHERAPEUTICS AND THEIR TARGETS

Human intestines house approximately 10^12^ lymphoid cells per meter and is known to have the highest density of immune cells in the body.^5^ Thus, this tissue provides an attractive target for different therapeutics that can modulate immune‐related diseases including cancer, autoimmune diseases, and infection.

Since the GI tract is inherently tolerance‐inducing, it also provides the opportunity to generate tolerance toward molecules that the body has not been exposed to previously. For example, intravenously delivered therapeutics such as checkpoint inhibitors^6^, ^7^ or anti‐VEGF^8^ can generate neutralizing antibodies, called antidrug antibodies,^9^ which severely reduce their efficacy. Therefore, generating strategies for presentation of these therapeutics to the oral or mucosal immune system provides a unique opportunity to generate tolerance toward intravenously infused drugs, and thus improve efficacy by diminishing the generation of antidrug antibodies.

Although, GI tract is naturally tolerance inducing, it is also the site of entry for most of the pathogens, and developing immune responses that can eliminate these pathogens in the tolerance‐inducing environment is especially challenging. Therefore, generating DDS that can orally deliver therapeutics (e.g., vaccines) targeted toward immune cells such as dendritic cells (DCs) can be highly beneficial. This topic will be discussed in detail later in this review.

In addition to vaccines, another important class of immunotherapeutics are interleukins (ILs, e.g., IL‐10, IL‐4, IL‐2), which can have dramatic effect on immune responses in the gut. These ILs have their specific receptors on different immune cells that line the intestine, and thus provide druggable targets for generating immunotherapy, which can be either pro‐ or anti‐inflammatory. In addition to ILs, growth factors can also generate robust immune responses by proliferating specific type of immune cells. For example, granulocyte macrophage colony stimulating factor (GM‐CSF) can be utilized to proliferate innate cells (e.g., DCs), ^10^, ^11^ and is utilized in clinic for treatment of cancer.^12^ Targeting GM‐CSF specifically to colon tumors can be achieved by developing DDS that deliver this growth factor at the site of lesion, thereby making the therapy more effective. Moreover, small molecules such as rapamycin^13^, ^14^ that target the mTOR pathway^15^ if targeted to specific sites of the immune system within the gut can provide site‐specific immune suppression. Lastly, delivery of specialized probiotics in the GI tract that can modulate the immune function is another major area where DDS can make a large impact. Some of the strategies that DDS utilizes to deliver these immunotherapeutics are discussed in this review.

Despite tremendous promise of DDS there still exists natural GI tract barriers that have to be overcome if oral delivery is to be considered for immune engineering. These natural barriers are briefly discussed below, and for further information on this topic readers are encouraged to read more specialized reviews. ^1^, ^16^

## 3 GI TRACT BARRIERS THAT PREVENT ORAL IMMUNOTHERAPEUTIC DELIVERY

### 3.1 Physiochemical barrier

Orally delivered pharmaceuticals travel through the upper (mouth to the duodenum of the small intestine) and lower (most of the small intestine to the large intestine) segments of the GI tract, and the latter segment contains the most barriers for oral delivery yet houses most of the drug absorption. As the drug travels through the upper segment of the GI tract, it encounters the degradative environment of the stomach (pH from 1 to 3) and is also met by strong proteolytic gastric enzymes (i.e., lipase, pepsin, amylase). ^17^ This acidic environment and increased proteolytic activity within the upper GI tract can lead to the degradation of drugs before they reach the small intestine for absorption, therefore, limiting the efficacy of the drug. ^18^ Furthermore, these pharmaceuticals must also be able to overcome mechanical stress (gastric flow) that resist the progression of the drug.^18^ Notably, proteins and other large biologics, like immunoglobulins, undergo stability and absorption challenges due to rapid degradation in the gut. ^18^ In one study, bovine milk immunoglobulin exhibited a 96% reduction in its rotavirus‐neutralizing activity in vitro when incubated with pepsin at a pH of 2, thus demonstrating the consequential effects of the GI environment on large biologics.^19^ Therefore, it is important that biologics must be specially modified to endure the natural characteristics of the gut.

Additionally, orally delivered drugs also have reduced systemic availability as compared to drugs that are delivered intravenously or intranasally due to the phenomena known as first‐pass metabolism. ^20^ The first‐pass effect describes how the concentration of an orally administered drug is reduced prior to meeting systemic circulation due to decreased gastric residence time and enzymatic degradation.^20^ Kolars et al. demonstrated this effect in their study, where cyclosporin was delivered to the small bowel of two patients following liver transplantation.^21^ Approximately 25% and 51% of total cyclosporin‐derived metabolites were observed in portal blood for the patients after 60 min of delivery, thus indicating heightened metabolic degradation of cyclosporin.^21^ This reduced availability of the drug in the systemic circulation directly decreases the sustained response that the oral therapeutics were initially intended to produce. The reduction of drug available in the systemic circulation is likely attributed to the drastic physiochemical conditions existent in the GI tract, such as the steric barriers of the mucosal immune system and the physical barrier of the intestinal epithelial layer.^18^, ^20^ In order to overcome the first‐pass effect, oral drugs are typically administered at a larger concentration; however, this then affects the toxicity and efficacy of various pharmaceuticals. ^20^

### 3.2 Epithelial barrier as immune defense

**FIGURE 1:**
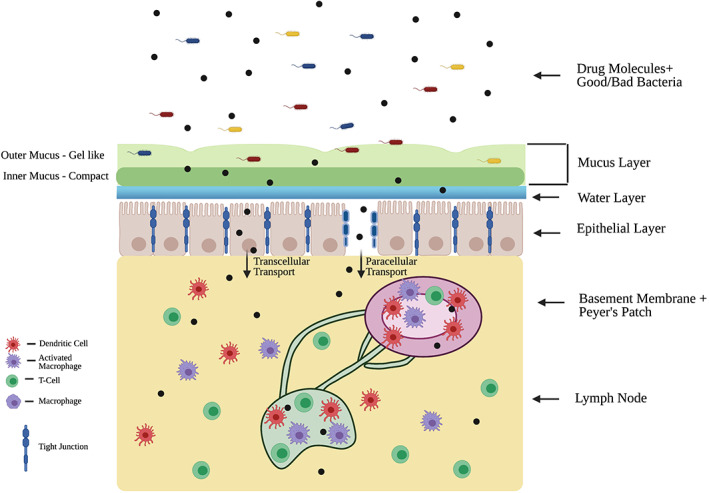
Drug molecules must bypass various barriers in the intestinal tract to reach systemic circulation. Some of these barriers include microbiota, mucosa, epithelial cells, and the immune system. Microbiota maintain immune homeostasis in the gut. A double‐layered mucosa coats the epithelium, which is bound by tight junctions. The basement membrane forms a dense layer under the epithelial layer. The gut barrier also houses several key immunological components (Peyer's patches, lymph nodes, dendritic cells, macrophages, T cells), which play an important role in preventing foreign pathogens/materials from invading systemic circulation

Immunotherapeutics must also overcome the challenge of limited traffic time in the GI tract and limited surface available for absorption (Figure 1, all schematic figures generated using Biorender.com, unless otherwise stated).

The epithelial layer of the GI tract contains tight junctions that further regulate the movement of substances within and through this surface, forming the first line of defense of the immune system.^22^ These tight junctions create a barrier that affects both the paracellular and transcellular transportation of molecules through epithelial tissue, thus molecules attempting to reach systemic circulation must cater to the underlying mechanisms of active/passive transport through this layer.^23^ Encountering these challenges therefore reduces gastric residence time of a pharmaceutical and adds to the overlying challenge of delivering a sustained effect of orally administered drugs.

**FIGURE 2:**
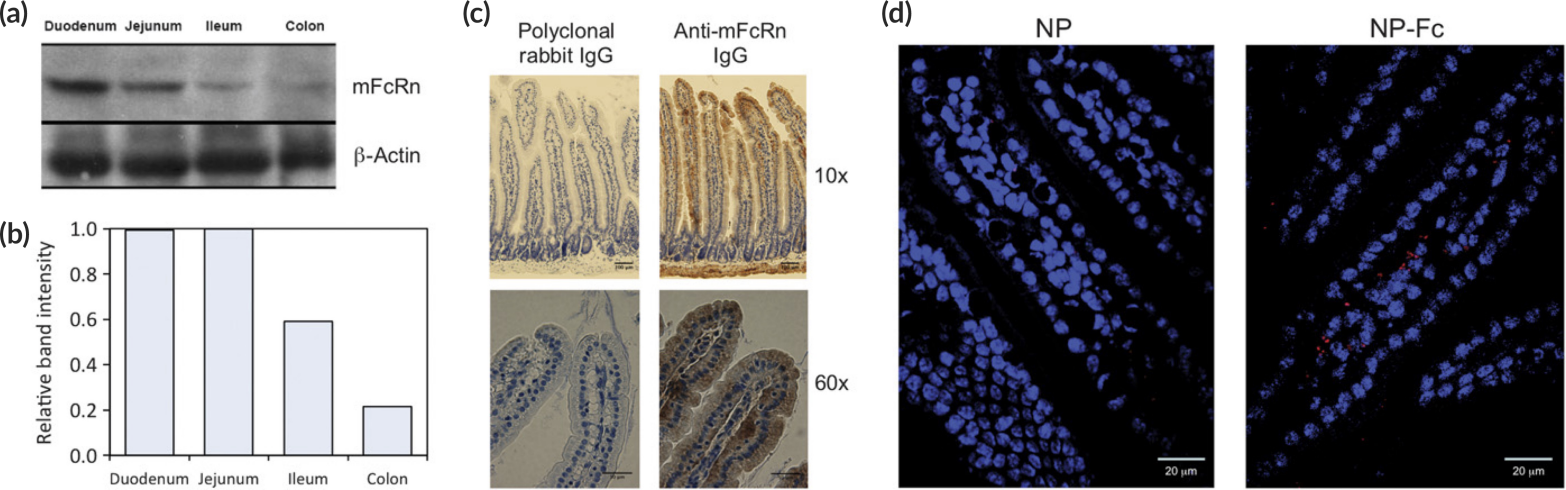
Nanoparticles conjugated with IgG Fc can be targeted to the intestinal epithelium. (a–c) FcRn is expressed in different parts of the intestines. (d) Nanoparticle intestinal uptake in mice shown here in red *Source*: Reprinted/adapted from Pridgen et al. *Sci Transl Med*. 2013;5(213):213ra167, © The Authors, some rights reserved, exclusive licensee AAAS. Distributed under a CC BY‐NC 4.0 license http://creativecommons.org/licenses/by‐nc/4.0/

Notably, epithelium that act as a defense mechanism can also be utilized to deliver drugs to the immune system as well. For example, Pridgen et al. demonstrated that polyclonal IgG Fc conjugated with poly(lactic acid)‐polyethylene glycol nanoparticles could be utilized to target the Fc receptor (FcRn) presented by the epithelial cells. Moreover, this study showed that these nanoparticles conjugated to IgG Fc were able to transcytose through the epithelium in vitro. ^24^ Lastly, this study also showed that orally administered FcRn‐targeted nanoparticles increased he mean absorption efficiency of the nanoparticles ~10‐fold as compared to the control of nanoparticles that were not targeted to FcRn (Figure 2). ^24^ This strategy of utilizing FcRn to target the epithelium can be utilized to generate immunity against infectious pathogens (e.g., M72 antigen against Tuberculosis), but also toward generating tolerance in autoimmune diseases (e.g., collagen for treatment of rheumatoid arthritis).

### 3.3 Intestinal microbiota

Microbiota consists of microorganisms that reside in the gut of mammals and are necessary for the maintenance of immune homeostasis in the gut.^25^ Gut microbiota act in a mutually beneficial relationship with the host to both strengthen the immune system through a series of microbiota‐dependent cascades within the epithelium as well as allow microbiota to thrive in the mucus.^26^ However, such microbiota can still serve as a threat if the immune system is weakened.^26^ Furthermore, it is important to note that while the GI tract serves an essential function in the digestion of foods through the existence of specific characteristics within its dynamic environment and complex regulative mechanisms, it can also impair the efficacy of orally administered pharmaceuticals.^25^, ^26^

**FIGURE 3:**
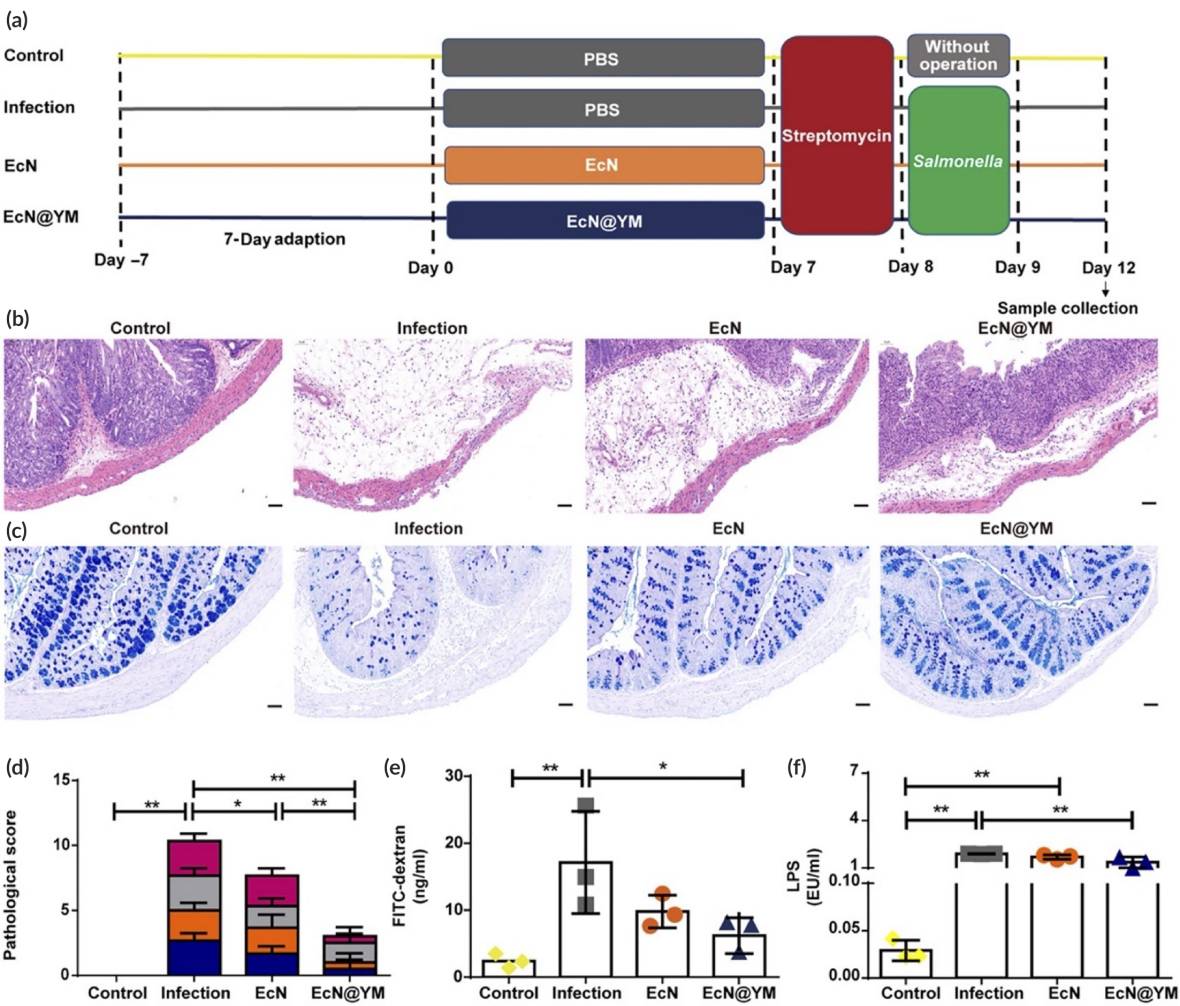
Yeast membrane coated *Escherichia coli* Nissle 1917 (EcN@YM), prevents intestinal barrier impairment from *Salmonella* infection. Using the experimental design (a) this study shows that EcN@YM prevented Salmonella mediated submucosal edema (b), depletion of goblet cells (c), pathological score (d), and an increase in intestinal permeability (e and f) *Source*: Reprinted/adapted from Lin et al. *Sci Adv*. 2021; 7(20):eabf0677. © The Authors, some rights reserved, exclusive licensee AAAS. Distributed under a CC BY‐NC 4.0 license http://creativecommons.org/licenses/by‐nc/4.0/

Interestingly, microbiota which prevent the drug permeation through the gut, also can be used as a therapeutic themselves for immunotherapies. For example, Lin et al. demonstrated that a probiotic, *Escherichia coli* Nissle 1917 (EcN) can be delivered to the Peyer's patches that can then induce anti‐inflammatory responses in the intestine (Figure 3). Interestingly, this study took advantage of β‐glucan embedded on yeast membrane to target the M cells, by coating EcN with yeast membrane. ^27^ Importantly, this study demonstrated that delivery of yeast membrane coated EcN (EcN@YM) when delivered orally, could localize to the Peyer's patches (immune organ), where they can generate an immune response to prevent degradation of the intestinal barrier. ^27^

### 3.4 Mucosal immune system of the gut

Among the first lines of immunological protection in the GI tract is the specialized mucosal layer that lines the surface of the epithelium. The mucosal layer is a gel‐like structure composed of glycoproteins called mucins which are secreted by goblet cells that line the intestine.^28^ Unfortunately, the constant production of mucus in the GI tract also largely reduces the availability of orally administered therapeutics to their targets. The mucosal system functions as a specialized immune defense system of the GI tract by detecting luminal foreign entities and either removing or neutralizing them while protecting the body's natural microbial flora.^29^, ^30^ Interestingly, mucosal surfaces vary in thicknesses along the GI tract due to the structure of their charged glycoproteins. This forms a steric barrier that restricts movement throughout its layer and drugs must permeate the mucosal barrier before entering systemic circulation. ^31^ Mucus is constantly being secreted and cleared quickly, thus trapping and removing foreign structures jointly and decreasing residence time of delivered drugs.^31^ The gut mucosa poses a particularly significant challenge for immunotherapeutics, such as antibodies and other large proteins, due to its dynamic nature and steric barriers. Particularly, it was shown that the diffusion coefficient decreased with increasing molecular weight of proteins when tested in vitro in porcine intestinal mucus, thus demonstrating the size‐dependent barricade of the steric mucosal barrier.^32^ Large proteins, especially antibodies, are also found to bind with mucins through electrostatic forces or strong hydrogen bonds thus essentially being trapped and unable to reach systemic circulation.^33^, ^34^ Therefore, for developing oral to systemic immunotherapeutics it is necessary to design them so that these are able to overcome the mucosal barriers.

**FIGURE 4:**
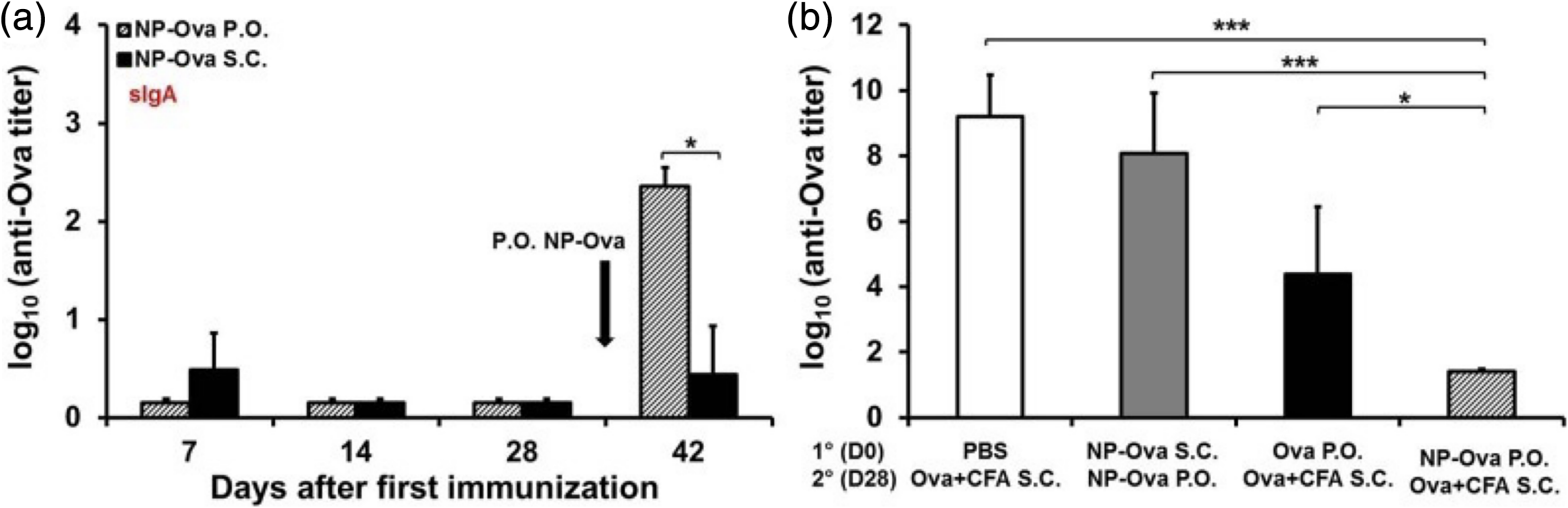
Induction of intestinal IgA and serum IgG2c antibody response depends on the immunization route that was used for priming *Source*: Reprinted/adapted from Howe et al. *PLoS One*. 2015;10(2):e0118067, with permission from Creative Commons Attribution (CC BY) license, PLOS One

Notably, Howe et al. demonstrated that not only the route of administration (systemic vs. oral) of therapeutics but also whether the therapeutic is associated with a nanoparticle is important for the development of an immune response. Specifically, this study demonstrated that oral delivery of ovalbumin (OVA) protein alone induce tolerance, whereas OVA conjugated to nanoparticle induced immunogenic response (Figure 4). ^35^ Moreover, subcutaneous boosting with OVA further increased the production of IgA titer, which is important for developing immunity against oral pathogens. ^35^ These data suggest that the mucosal immune systems barriers can be taken advantage of depending on the type of immunity desired (tolerogenic versus immunogenic). Therefore, stronger IgA‐based immunity against mucosal infections such as SARS‐COV2 may be generated by not only immunizing in the non‐mucosal tissue (e.g., intramuscular injections) but also orally with appropriate adjuvants.

The commensal bacteria reside primarily on the outer mucosal layer and actively interact with the mucin. For example, *Bacteroides thetaiotaomicron*, a type of commensal bacteria, has been shown to increase goblet cell differentiation, which are then responsible for generating mucin. ^36^, ^37^, ^38^ Different probiotic agents, which function to grow and restore intestinal flora, have also been found to stimulate mucin protein production and thus help to enhance pathogenic resistance.^39^, ^40^ Therefore, it is evident that commensal bacteria play a significant role in maintaining immunity at the mucosal layer. It is important to note, though, that the success behind this symbiotic relationship is still being widely investigated and conflicting evidence suggests that the penetration of commensal bacteria through the mucosal layer is associated with inflammatory diseases, such as inflammatory bowel disease or Crohn's disease.^41^

Although the outer mucosal layer houses various microbiota that live in a symbiotic relationship with the host, the inner mucosal layer is nearly devoid of any bacteria.^42^ Structurally, the inner mucosal layer is more compact than the outer layer, and thus serves more as a physical line of defense against pathogenic agents or foreign entities.^42^ Drug delivery vehicles, therefore, must also seek new pathways to combat against the constantly recycled inner and outer layer of mucus.^43^ This may involve coating the drug carrier with a specialized polymer that allows for increased mucosal penetration or adhesion.^31^, ^44^, ^45^

Because the intestinal mucus provides a niche for various types of microbiota, it is important that the intestinal immune system be able to differentiate between commensal bacteria and pathogenic bacteria. The exact method in which a homeostatic environment is maintained is still unknown, though various studies have shown that the presence of antigen‐presenting cells, specifically DCs, along with assorted populations of B and T cells within the mucosa are likely related to the discrimination between commensal and pathogenic bacteria.^46^ The subset of DCs found in the mucosa have been compared to similar DCs found in the respiratory tract where they play a notable role in tolerance, and thus building a specific tolerance toward commensal bacteria.^47^ Aside from commensal bacteria, the specialized immune system in the mucosa can also recognize certain food antigens to help prevent immune responses against the food antigens.^46^ Since the mucosa and the intestinal immune system are so tightly related, malfunctions at the mucosal layer, where immune responses can be triggered against non‐pathogenic bacteria, are the general basis for intestinal inflammatory diseases, such as Coeliac disease or Crohn's disease.^46^ However, this relationship between the mucosa and the intestinal immune system demonstrates the opportunity to target the mucosal layer for oral delivery of drugs. Moreover, targeting DCs or mimicking the commensal bacteria can be an alternative approach to deliver immunotherapeutics orally to the mucosal immune system and potentially treat these inflammatory diseases.

An understanding of each of the above discussed challenges is necessary to advance the construction of novel biomaterials to tackle specific barriers in oral administration. Oral DDS takes into account these barriers in order to improve drug availability for oral to systemic delivery. Few of the strategies to achieve this include modulating the epithelial barrier for drug absorption, formulating therapeutics to better adhere to the mucosal layer, or targeting specific immune cells in the gut (Figure 1). These DDS strategies are further discussed in detail in the following sections with examples expanding the potential application of these DDS strategies on immune engineering and immunotherapeutic delivery via oral route.

## 4 MUCOADHESION TO IMPROVE DRUG DELIVERY

**FIGURE 5:**
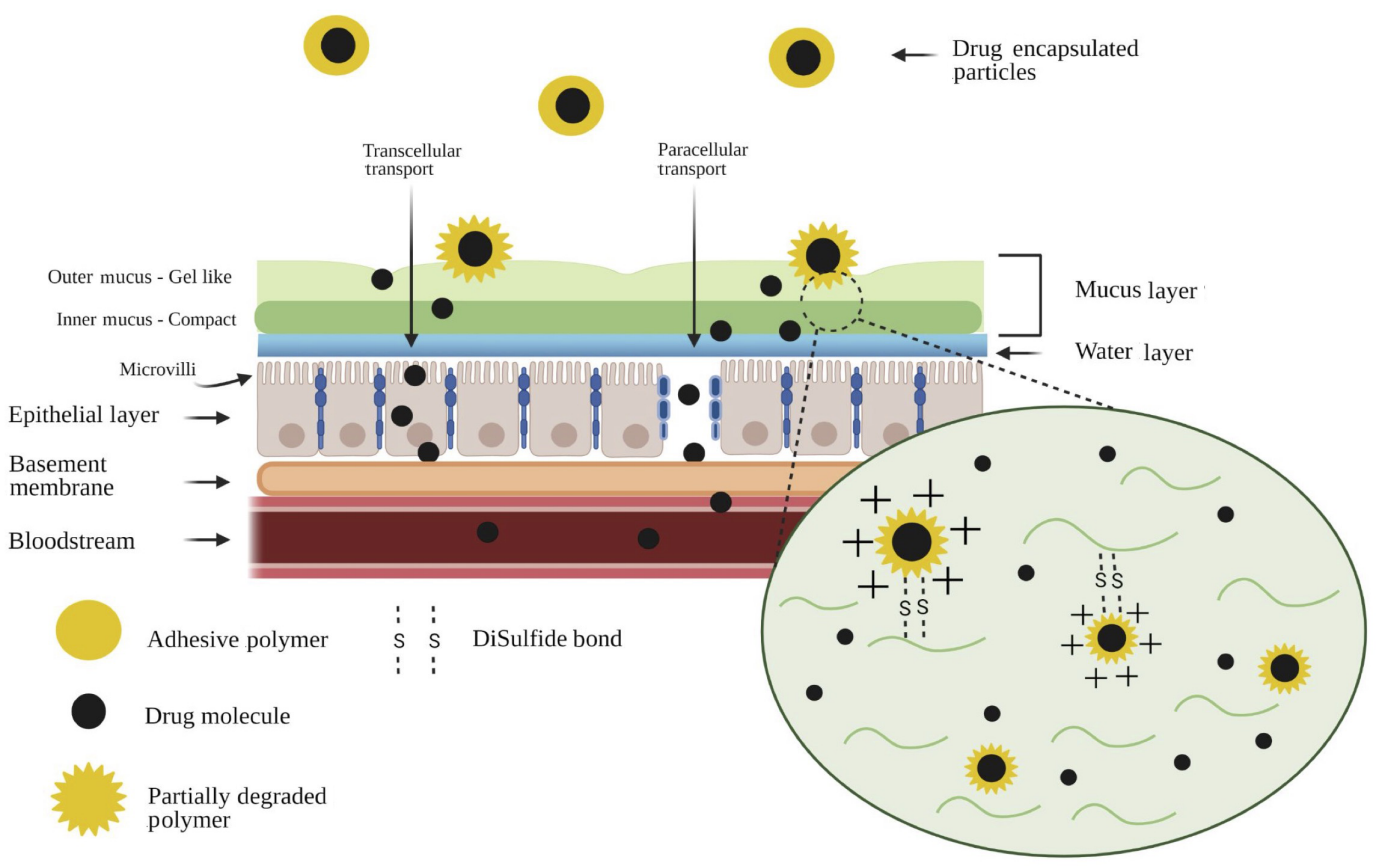
Mucoadhesive polymer coating on drugs can provide higher residence time in the gut. These mechanisms include disulfide bonds and ionic interactions with the mucosa

To optimize the amount of drug that is absorbed in the body, it is imperative that the carrier either releases a large amount of its contents in a short amount of time or releases a known amount throughout a set time. Currently, there is larger focus in the former, but recent developments of novel biomaterials have utilized mucoadhesion to increase the residence time of the drug in the GI tract. This method can enhance the therapeutic effect by increasing the absorption at the target site and can often be combined with enteric polymers (e.g., Eudragit) for gastric resistance and GI targeting.^48^ This effect was demonstrated in an in vivo study with diabetic rats using orally administered insulin enterically coated with Eudragit as well as a polymeric mucoadhesive layer consisting of polycarbophil–cysteine that showed a sustained decrease in blood glucose over a time period of 80 h before steadily reaching its initial value again. Moreover, no significant effect was observed when insulin was orally administered without the polymeric coating.^49^ Oral delivery techniques such as these, show promise in developing oral DDS that may allow for an increased gastric residence time with a controlled and sustained release of the orally delivered pharmaceutical (Figure 5). Mucoadhesion utilizes the formation of chemical bonds, most commonly hydrogen or ionic bonds, or even stronger covalent bonds between the mucosa and the mucoadhesive materials to prolong the residence time of absorption in the GI tract.^48^ In addition to expanding the absorption window of an orally delivered drug, formation of these chemical/ionic bonds in combination with specialized polymers can enhance permeation and prevent degradation of delivered agents. Some of the polymers that can achieve this include chitosan; however, this bond is not strong enough on its own to sustain the mucoadhesion and thus only slightly increases the residence time. ^50^ However, when mucoadhesive technologies are paired with thiolated polymers, mucoadhesive properties can dramatically increase, as shown in an in vitro study demonstrating that thiolated polymers showed significant stability as opposed to non‐thiolated polymers and did not exhibit any disintegration behavior over the observation period of 48 h.^51^ Thiolated polymers interact with the mucosal layer to form strong covalent disulfide bonds (Figure 2) that promote a more structurally stable carrier, in turn increasing the residence time in the GI tract at the target location.^32^ In another study, thiolated chitosan micelles demonstrated up to a 56‐fold higher degree of attachment to intestinal mucosa compared to unmodified chitosan micelles, thus demonstrating the potential of thiolation for improved mucoadhesion and drug delivery.^52^

The strategies of mucoadhesion can be utilized to increase residence time of immunotherapeutics such as cytokines and growth factors. Interestingly, diseases such as Ulcerative colitis and Inflammatory Bowel Diseases may benefit from local delivery of anti‐inflammatory cytokines such as IL‐10^53^ and IL‐27, ^54^ and antibodies such as anti‐IL‐22. ^55^ The extended residence of these therapeutics will ensure reduction of inflammatory cascade induced by pro‐inflammatory cells (e.g., T helper type 1 and T helper type 17) in the intestine, and provide immune homeostasis.

Interestingly, Chung et al. reported that orally delivered IL‐10 releasing poly(lactic acid) microparticles ameliorated local GI polyposis in mice. This study was able to demonstrate that these particles were taken up in the Peyer's patches, which could then polyp numbers and anemia in mice significantly as compared to the control. ^56^ Importantly, this study demonstrated that these particles could increase the survival of mice significantly as compared to controls. Therefore, delivery of anti‐inflammatory cytokines can be a powerful tool to locally modulate the immune response to address chronic diseases.

Another strategy for overcoming the mucosal barrier includes nanoparticle systems that use specialized mucolytic agents. These are conjugated on the surface of the particles and have the ability to cleave mucus substructures, which then allows for the drug carrier to bypass the mucosal layer.^52^ De Sousa et al. examined papain (PAP) and bromelain (BRO) as mucolytic agents, both of which were shown to permeate through nine 2 mm intestinal mucus gel segments while the unmodified nanoparticles were found to only permeate through the first few segments.^57^ The diffusion coefficient of intestinal mucin also exhibited a 2‐fold increase in the presence of PAP and BRO as opposed to unmodified nanoparticles.^57^ These results demonstrate the ability of mucolytic agents to permeate the mucosal layer in the GI tract. Since both the PAP and BRO enzymes are digested in the gastric environment, utilizing enteric coatings would be one idea to consider for the purpose of ensuring the enzymes are not degraded by the harsh gastric acidity for the successful delivery of immunotherapeutics.

The combinatorial delivery of multiple targeting mechanisms allows for the strength of one agent to compliment the strength of the other for an effective therapeutic delivery. For example, self‐nanoemulsifying drug delivery systems (SNEDDS) as drug carriers were designed to increase drug dissolution and solubility due to their lipophilic nature.^58^ Interestingly, SNEDDS cannot adhere to the mucosal layer within the GI tract due to the net‐negative charge of the mucus. An overall positive charge can be generated if SNEDDS are paired with mucoadhesives (e.g., chitosan derivatives) for binding to the negative mucosal layers, and thus increasing availability of drugs with poor solubility in these environments. The slightly negative charge of the mucosal layer explains the partial ionic binding of the positively charged acyl chitosan to the mucosal layer. This novel system has found success with drugs like saquinavir^59^ in mice experiments as well as tipranavir^60^ and cyclosporin A,^61^ which were found to have enhanced effects with orally delivered drugs as compared to the control in clinical trials.^62^, ^63^ An interesting application of this combination strategy of SNEDDS can be delivery of anti‐inflammatory agents for chronic autoimmune diseases such as type 1 diabetes, multiple sclerosis, and rheumatoid arthritis. For example, rheumatoid arthritis patients are required to inject themselves with anti‐TNFα every few weeks to limit the damage by immune cells to the tissues. Therefore, a strategy that allows for antibodies to be taken orally will allow for delivery of anti‐inflammatory agents from oral to systemic route, thereby potentially increasing compliance as well. However, after achieving mucosal penetration, the drug then must overcome transportation through the epithelial barrier before these immunotherapeutics can be delivered systemically, and different methods to overcome this barrier are discussed below.

## 5 OVERCOMING THE PHYSICOCHEMICAL CHALLENGES OF THE EPITHELIAL BARRIER

Development for the enhancement of drug delivery through the epithelia is largely dependent on the mechanism of permeation across the epithelial barrier. These primarily consist of simple passive diffusion, carrier‐mediated diffusion, active transport, and transcytosis initiated by epithelial cell‐mediated endocytosis.^64^, ^65^ Several studies provide evidence for simple passive diffusion as the primary mechanism of drug permeation.^66^, ^67^, ^68^, ^69^, ^70^ Simple passive diffusion involves the law of diffusion in which a molecule moves from an area of higher concentration to that of lower concentration and while this may be the most efficient mechanism since it does not require an energy input, many complications can arise due to varying properties of drug molecules, such as size and charge.^65^ Carrier‐mediated diffusion is also another common mechanism of drug absorption and utilizes a transmembrane carrier to transport a drug molecule across the epithelia.^65^ Active transport and transcytosis are not as common as passive diffusion and are significantly more energy‐expending processes. While active transport is not common for most therapeutics, some examples include levodopa^69^ for Parkinson's disease or fluorouracil^70^, ^71^ for cancer.

Transport of these therapeutics is further supported by the microstructure of the epithelial barrier, namely villi and microvilli. Since the villi and microvilli are finger‐like projections that extend off the epithelial barrier along the length of the intestine on the apical side facing the intestinal lumen, it helps to increase the amount of surface area that is available for absorption (Figure 5). Moreover, polarized regions on the apical surface form a specialized network that allows for sorting and packaging of materials in and out of the cell, which is particularly important for transcytosis and delivery of drugs to systemic circulation.^72^ Indeed, this process is the primary method for absorption of many immunoglobulins^73^ and proteins. ^74^

In addition to villi and microvilli, other microstructures of epithelial cells are to be taken into consideration when designing DDS for therapeutic delivery. For example, tight junctions within the epithelial barrier make it difficult for large drug molecules (≥6 nanometers) to pass through the epithelial layer.^75^ Two principal routes of absorption through the epithelial layer are the transcellular route and the paracellular route (Figures 1 and 5). The transcellular method involves transportation across the apical side of the epithelial cell membrane, transportation within the cell, and subsequent removal at the basolateral side of the epithelial cell.^76^ On the other hand, paracellular route involves permeation of the drugs between the cells.^77^ Two main characteristics of the drugs that determine permeation through paracellular route include the charge of the drugs and their size.^75^, ^78^

In order to utilize the mechanisms of paracellular drug delivery, strategies have been designed to enhance absorption and permeation between the cells. This process entails temporarily breaking down the epithelial cell membrane barrier or opening up intercellular tight junctions.^79^ Surfactants have been extensively researched for their ability to open epithelial tight junctions for drug permeation. Because surfactants are amphiphilic, the hydrophilic and hydrophobic components can align themselves at the epithelial interface in order to decrease surface tension and thus facilitate the transportation of molecules across the epithelial layer. Although surfactants, such as sodium dodecyl sulfate or polysorbate‐80 were found to increase drug absorption, they were also found to produce irreversible membrane damage.^78^, ^80^ Various animal studies have shown that transient permeabilizing agents are less cytotoxic when compared to agents that generate irreversible membrane permeation. Examples of such agents include ethylenediaminetetraacetic acid (EDTA), which function as calcium chelators, or vehicles comprised of fatty acid chains such as caprate or laureate, which operate through modulation of filament interactions in the membrane.^81^ Recently, negatively charged nanoparticles have also been found to enhance membrane permeability apparently with very little toxicity or permanence.^82^

As opposed to surfactants, small and negatively charged nanoparticles work by enhancing transcellular permeation. Regardless, permeabilizing agents that disrupt the integrity of the cell membrane also allow for the opportunity of solutes, other than the targeted drug, to pass through the membrane, thus compromising clinical implementation of such agents. Nevertheless, clinical trials have utilized these agents and found success through the combination of both paracellular and transcellular enhancements of permeation. For example, GI permeation enhancement technology (GIPET) is a formulation that is being developed by Merrion Pharmaceuticals that has found extensive success in clinical trials and has been pre‐approved per United States Food and Drug Administration (U.S. FDA) standards.^83^ The GIPET formulation utilizes fatty acid compounds to enhance membrane absorption and, in clinical trials, has been shown to increase the oral bioavailability by 12‐fold.^84^ Other permeabilizing agents that have found clinical success include Chiasma's transient permeability enhancer (TPE) technology, which increases the GI absorption of large macromolecules, shown through clinical trials with Octreolin for acromegaly,^85^ or Oramed Pharmaceutical's formulations of combining the delivery of enteric coatings and permeation enhancers (e.g., EDTA) to amplify absorption of orally delivered insulin.^83^ The success of these technologies is likely attributed to the utilization of transient permeabilizing agents that both work to increase drug absorption while also working rapidly to reverse any structural effects and minimize any extensive damage to membrane function.

In addition to synthetic surfactants, Zonula occludens toxin (Zot) coatings have also been studied for manipulating tight junction openings in the intestine for paracellular delivery. In vitro studies in rabbit ileum with Zot shows that the effects of pharmaceuticals delivered orally peaked at around 80 min and with reversible effects in a time‐dependent manner, which demonstrates the reversibility potential for Zot coatings for oral drug delivery.^86^ The mechanism behind tight junction manipulation by Zot involves a rearrangement of the epithelial cytoskeleton induced by a series of complex protein kinase C‐dependent signaling cascades.^87^, ^88^ Notably, Zot works similarly to zonulin, a protein abundant in the digestive tract that plays a significant role in tight junction regulation, both of which bind to the same receptor on intestinal epithelial cells, suggesting that this may be the reason for the effectiveness of Zot in epithelial permeation.^88^ Importantly, these studies utilizing chitosan and Zot coatings reported no apparent toxicity.^50^, ^86^ However, further research needs to be done to further elucidate this mechanism and analyze long‐term organ‐level and organism‐level toxicity in larger animal models.

**FIGURE 6:**
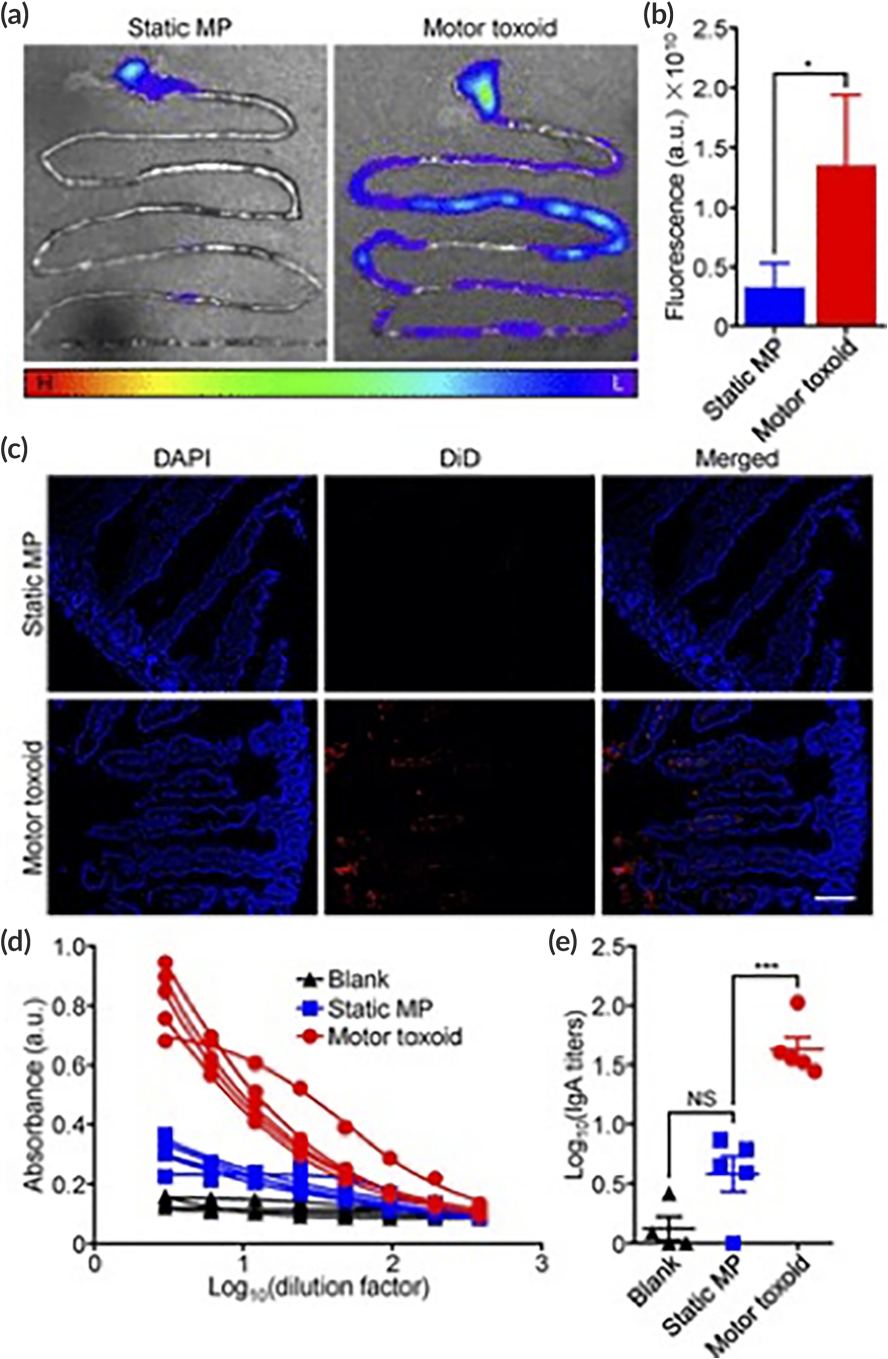
Delivery of Staphylococcal α‐toxin via “motor”‐based microparticles vaccines, enhanced distribution of the payload throughout the intestine (a–c), and lead to increase in IgA titers in mice (d and e) *Source*: Reprinted/Adapted from Wei et al. *Nano Lett*. 2019;19;1914–1921, with permission from Copyright 2019 American Chemical Society

This enhancement of active transport of drugs systemically from oral route can be highly beneficial for treating acute immune system‐related diseases, such as infections. In fact, targeting the mucosal immune system can be highly beneficial in generating immune responses against pathogens. For example, Wei et al. demonstrated that molecular‐motors consisting of a magnesium‐based core, can be utilized to deliver vaccines in the gut, by actively transporting the vaccine components into the intestinal tissue (Figure 6). In fact, they demonstrated that the active transport due to the “motor” effect of the particles allows the payload to be distributed uniformly throughout the intestinal length. ^89^ These particles were also able to significantly increase IgA antibody titers in the feces as compared to the controls, which indicates that active transport of vaccines via motor‐based particles led to higher immune response. ^89^ Therefore, this strategy of active transport might be beneficial to study mucosa‐related infections, such as Listeria and *Helicobacter Pylori*, where local short‐term generation of immune responses can have a profound effect on disease outcomes.

In addition to temporarily opening tight junctions, it is also possible to increase passive transport using prodrugs strategies. Specifically, drugs are chemically modified to attribute them the properties of enhanced passive transport. Prodrugs are administered as inactive substances and converted to its pharmaceutically active form when metabolized with favorable physicochemical conditions in the body.^76^ Prodrug design entails attaching of hydrophilic groups to enhance drug solubility and using lipophilic molecules to increase passive diffusion through the epithelial cell membrane.^90^ Design of prodrugs must also be specific to particular properties of the pharmaceutically active drug, as some prodrugs, if prematurely activated before or during diffusion through the epithelial membrane, can cause trapping of the active drug within the cells of the membrane, and thus not be able to produce the full effect at the targeted site. For example, the L‐valyl prodrug of zanamivir (antiviral drug) was shown to improve epithelial cell permeability with around a 3‐fold increase in absorption as opposed to the acyloxy ester prodrugs that inhibited uptake.^91^ Other mechanisms of prodrug design include targeting transporters located on both the apical and basolateral side of intestinal epithelial cells to allow for enhanced drug absorption. The human peptide transporter 1 (PEPT1) based prodrugs are an example of targeting specific uptake transporters that show promise for oral drug delivery.^90^

In another study, it was found that oral delivery of insulin with the cell penetrating peptide (CPP) penetratin showed up to a 78.6‐fold increase in its hypoglycemic effects, lasting up to 18 h, as compared to the insulin control, giving a pharmacological availability of 18.2%.^92^ Additionally, in situ experimentation found a dose‐dependent increase in insulin absorption in the ileum when administered with oligoarginine, another CPP.^93^ While the mechanism behind CPPs are not fully understood, a form of endocytosis has been proposed as the method of cell penetration and targeted antigen delivery.^94^, ^95^ Since insulin is a peptide, other immunomodulatory peptides and proteins can be potentially delivered using CPP for oral to systemic delivery; however, this area needs to be researched further, and is discussed in the following section.

## 6 CURRENT ORAL TO SYSTEMIC IMMUNOTHERAPEUTIC DELIVERY STRATEGIES

Copious research has been done that demonstrates the potential for various therapeutics that can be used to modulate the immune system against a host of diseases such as autoimmune diseases and cancer. ^96^, ^97^ Direct delivery to systemic circulation is usually obtained through intravenous administration; however, noninvasive routes of administration, particularly oral formulations, are especially advantageous in being cost‐effective and efficient methods of drug delivery for higher patient compliance. Immunotherapeutics can take many different forms; however, many promising agents, such as monoclonal antibodies and other relevant proteins, as well as genetically modifying agents, such as siRNA and mRNA, are easily degraded in the presence of gastric enzymes or impermeable to the complex layered GI immune system, and thus face decreased absorption and reduced drug effect with oral administration. In order to overcome the challenges attributed to oral administration, complex delivery systems and biomaterials have been, and continue to be, developed to further increase bioavailability and evolve this route into a more viable option. The focus of these delivery systems and biomaterial strategies specifically targets the physical and biological barriers en route for orally delivered drugs to reach systemic circulation.

**FIGURE 7:**
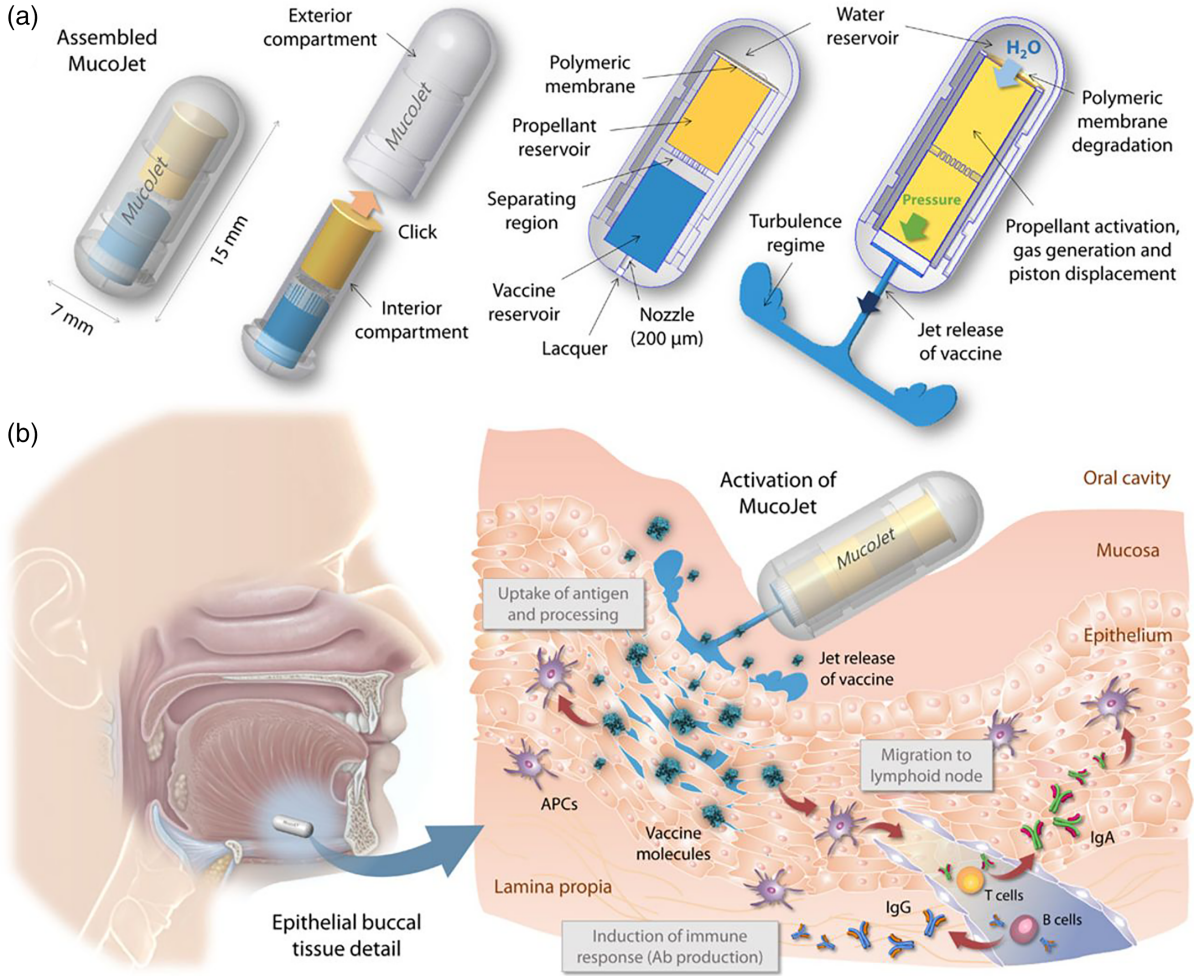
(a) The two components of the MucoJet are clicked together by the user prior to administration. The polymeric membrane is then dissolved. Water contact with the chemical propellant causes a chemical reaction that produces carbon dioxide gas, inducing a sufficient jet velocity and pressure for the vaccine solution to penetrate the buccal mucosa layer. (b) The MucoJet is delivered by mouth to buccal tissue (left). Upon penetration through the mucosal layer, the vaccine solution is delivered to antigen‐presenting cells (APCs) in the mucosa‐associated lymphoid tissue (MALT) to generate an immune response (right) *Source*: Reprinted/adapted from Aran et al. *Sci Transl Med*. 2017;9(380):eaaf6413. © The Authors, some rights reserved, exclusive licensee AAAS. Distributed under a CC BY‐NC 4.0 license http://creativecommons.org/licenses/by‐nc/4.0/

Natural polymers as well as synthetically formulated biomaterials have been developed to increase the time spent during absorption in the GI tract. As previously discussed, chitosan is an example of a natural polymer that increases the time of absorption for orally delivered pharmaceuticals. For example, pre‐clinical trials using chitosan‐based nanoparticles loaded with 10‐Hydroxycamptothecin (HCPT) have found success in increased cell uptake and drug absorption for immunotherapy of melanoma^98^ and has also been reported to enhance more efficient drug delivery of peptides, such as with eugenol‐loaded chitosan nanoparticles, which produced anti‐inflammatory effects in an aggressive model of rheumatoid arthritis.^99^ The MucoJet is another example of a novel technology that has found success with in vivo animal studies to penetrate the mucosal layer via oral administration and elicit antibody production.^100^, ^101^ The MucoJet is a small immunotherapeutic delivery system consisting of a small plastic device that can be activated by the user by providing a pressure termed “click” in their work (Figure 7).^101^ The polymeric membrane dissolves due to the “click,” causing the water reservoir to contact the chemical propellant (citric acid and sodium bicarbonate), generating carbon dioxide gas (Figure 7).^101^ This gas production increases the pressure to around ~30 kPa, providing the device with sufficient force to penetrate the mucosal layer and deliver a vaccine solution.^101^ Aran et al. utilized this technology to deliver ovalbumin to rabbits, which produced high titers of antigen‐specific immunoglobulins G and A.

Each of these mentioned strategies provide advanced progress in increasing the bioavailability of orally delivered drugs, thus, making it a more attractive means of drug delivery. Although each of these strategies have made considerable progress, the safety, cost, and clinical translation of these technologies still remain unclear. Therefore, it is particularly important to address specific challenges of oral administration with novel biomaterials to make this route a viable candidate for immunotherapeutic delivery.

In addition to oral to systemic delivery of immunotherapeutics, delivery of immunotherapeutics to the immune system of the mucosa has gained a lot of interest in recent years. Interestingly, mucosa is the site where most of the pathogens enter the body (>90%),^102^ and hence it can be advantageous to utilize immunoengineering technologies to deliver immunotherapeutics to the mucosal immune system.

## 7 TARGETING M‐CELLS FOR IMMUNOTHERAPEUTIC DELIVERY TO THE MUCOSAL IMMUNE SYSTEM

**FIGURE 8:**
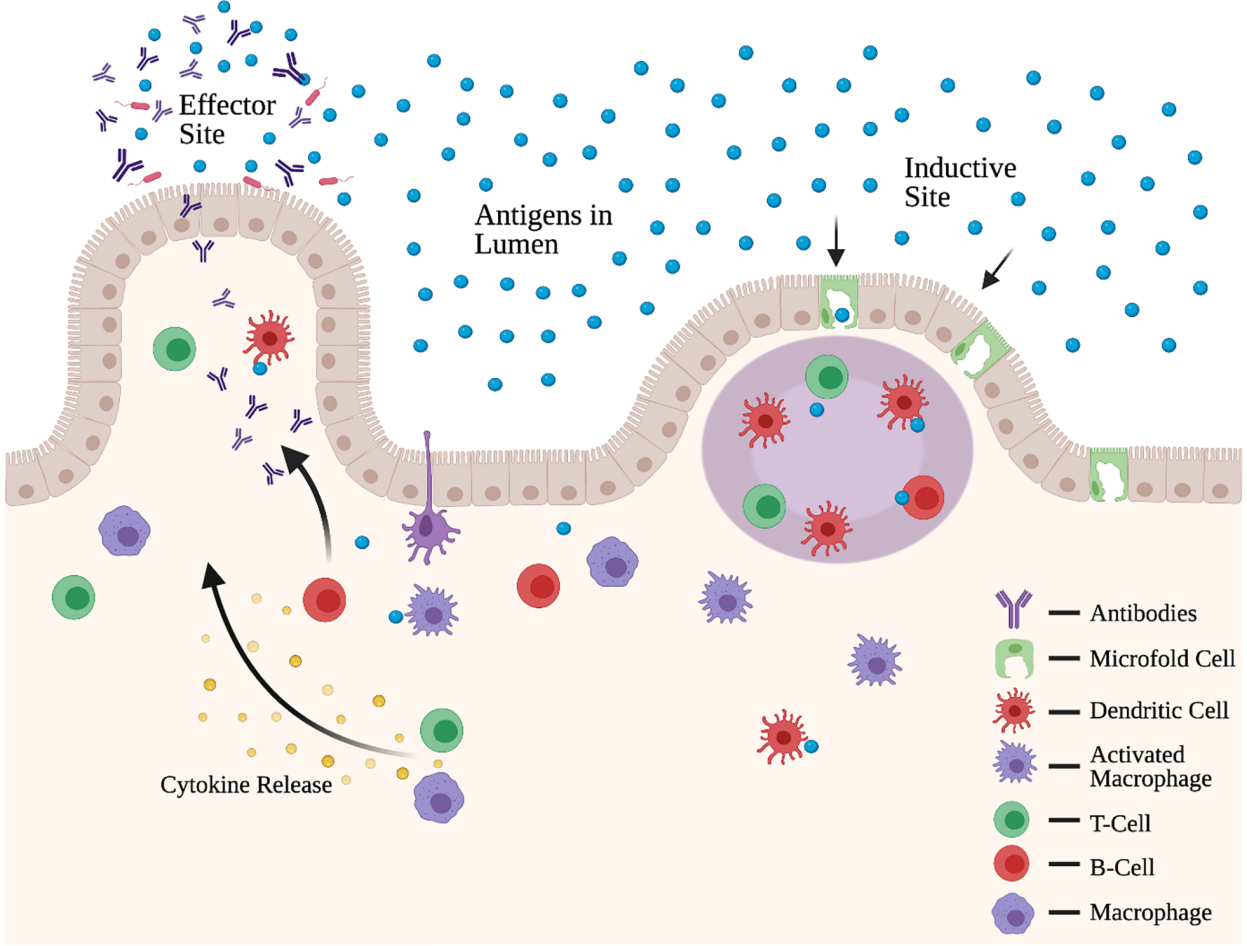
Inductive sites (right) made up of T and B cells within the Peyer's patches and effector sites (left) within the lamina propria comprise of intestinal immunity within gut‐associated lymphoid tissue. M‐cells along the epithelia allows for antigen uptake

Microfold (M) cells are specialized intestinal epithelial cells that are commonly found in mucosa‐associated lymphoid tissue (MALT) and in gut‐associated lymphoid tissue (GALT). In the case of GALT, these cells specialize in transporting antigens from the lumen of the small intestine toward lymphoid tissue.^103^ More specifically, at the inductive site, M cells transcytose antigens to be processed in the Peyer's patches, which is compacted with various immune cells, such as DCs, B cells, and T cells (Figure 8).^103^, ^104^ Upon activation, immune cells produce cytokines and altogether are involved in the release of antibodies for an antigen‐specific immune response (Figure 8).^103^, ^105^ Since M cells interact with these specialized cells responsible for generating a vaccine response, they are an ideal target for generating a vaccine response.^105^ Current potent examples of oral vaccines include rotaviruses, polioviruses, and cholera vaccines.^106^

Effective targeting of M cells for oral vaccine delivery can be challenging due to the structure of the cell and the environment in which they reside. M cells have the ability to uptake foreign entities and bypass the apical epithelial layer to directly deliver these foreign particles to the basolateral layer.^107^ Although M cells may be an ideal target for immunotherapeutic delivery, only 1 in 10 million epithelial cells in the GI tract are M‐cells.^108^ While it is possible to amplify the frequency of M cells, it might be advantageous to target existing M cells for efficient drug delivery.

Various animal studies have taken advantage of this efficacy for M cell targeting in inducing immunological responses for oral vaccination at the mucosal surface. For example, M cell targeting using a ligand reovirus protein sigma1 has been shown to facilitate oral tolerance in mice and thus exhibit the key role of antigen uptake and M cell targeting in mucosal immunity.^109^ Studies have also looked at the specific relationship between M cells and secretory immunoglobulin A (sIgA), which is one of the main defense mechanisms of the MALT. ^107^ sIgA plays a significant role in regulating immunity in the mucosal epithelial by binding and removing foreign antigens and pathogens that are found within the mucosal surfaces. Notably, sIgA is also associated with helping immune tolerance by binding to dietary antigens and organisms in the microbiota.^110^ Importantly, sIgA that is complexed with antigens, can also be endocytosed by M‐cells via reverse transcytosis.^111^, ^112^ This reverse transcytosis then provides the antigens directly to immune cells, such as DCs, which are present in the MALT.^111^ In addition to sIgA, studies have also exhibited success with M cell targeting by utilizing Claudin 4 targeting peptide (CPE) which was found to enhance mucosal IgA responses.^113^ Additionally, studies have also utilized p24 gag antigens linked to sIgA to elicit HIV‐specific immune responses.^114^

Plant lectins are another notable method used to target M‐cells. *Ulex europaeus agglutinin* 1 (UEA‐1) lectin binds specifically to the alpha1,2 fucose residue that is expressed on the apical surface of the mouse M‐cells.^115^ This selective binding promotes rapid uptake of antigens by M cells. Notably, the apical surface of the M cell faces the lumen, thus, making M cells an optimal target for oral vaccinations since the lumen is where oral vaccinations would be found. It is important to note, however, that a major drawback to utilizing plant lectins are their potential to produce antinutritional/toxic effects, such as those observed in studies showing significant weight loss in pigs after being fed *Phaseolus vulgaris* agglutinin (PHA), a kidney bean lectin.^116^

Plant lectins can also have an affinity for specific glycoproteins and, therefore, these can be utilized to target glycosylated proteins on M cells. Glycoproteins are located on cell surfaces to aid in immune defense and, due to their unique patterns and structures, can also serve as an identity marker. However, little is actually known about the structure and function of glycoproteins on M‐cells. Interestingly, M‐cells have a distinct glycosylation protein profile as compared to other localized cells which, in turn, provides a mechanism to differentiate M cells from its surrounding cells.^103^ Specifically, the glycocalyx, which is a form of glycolipid/glycoprotein coating that serves as a barrier between a cell and its surroundings, is thinner on M cells than the glycocalyx of its neighboring cells.^103^ The reduced glycocalyx on M cells adds to its overall unique structure and allows easier access to the intestinal lumen for more efficient uptake of antigens, thus making it a targeted region of interest for the improvement of immunotherapeutic delivery. Additionally, glycosylation proteins vary in different locations of the intestine and also differ between species. This can potentially be used to target specific locations in the small intestine, for a targeted delivery and localized drug activation. To date, only little is known about the types of receptors that exist on the surface of M‐cells for recognition and subsequent endocytosis, therefore, it is important to further explore how M‐cells can be targeted to uptake specific antigens for immune targeting while avoiding the absorption of toxic and invasive pathogens.

## 8 ORAL VACCINATION—STATE OF THE ART AND ROLE OF IMMUNOENGINEERING

Although there are more than 20 actively administered vaccines in the United States, only rotavirus, adenovirus, cholera vaccine, and oral typhoid vaccines are administered orally. ^117^ Currently, most vaccines are delivered by intradermal or intramuscular injections, which are associated with problems such as safety and high cost of mass immunization.^118^, ^119^ Unfortunately, vaccines administered either intradermally or intramuscularly, provide only partial, or in some cases, no protection at the mucosal site, where most (>90%) of the pathogens access the body.^102^ Therefore, targeting and generating mucosal immune responses against pathogenic proteins or self‐proteins for tolerance can be highly beneficial. Notably, the mucosal immune system tends to be immunosuppressive and, therefore, provides an attractive target for generating tolerance inducing vaccines as well. However, there are very few oral or intranasal vaccines available, and this can be directly linked to the lack of delivery systems capable of delivering proteins (antigen) and adjuvants (provides context for vaccines—immunogenic/tolerogenic) to the mucosal immune system.

Biomaterials, such as microparticle‐based systems, can target the GALT typically by introducing antigens to the inductive sites on the surfaces of tissues to streamline an immune response to the effector sites (Figure 6). As briefly discussed in the previous section, antigens that are transcytosed by specialized M‐cells are presented to antigen‐presenting cells (e.g., DCs, B lymphocytes, and macrophages) for the induction of immune responses.^120^ Producing a sustained immune response with mucosal vaccination by targeting DCs can be challenging, but have found success in mice studies through the manifestation of immunologic memory via directly inducing cytotoxic T cell activation.^121^

Another area where mucosa‐targeted vaccines can have a major impact is with autoimmune diseases, where tolerance against antigens of interest is desired. For example, in autoimmune diseases, such as rheumatoid arthritis (RA), the body mistakes self‐antigens (i.e., collagen in the case of RA) as foreign, which leads to immune responses being mounted against the self‐antigen. Specifically, delivery of antigens orally has been tested in clinical settings with mixed results, and no treatment has yet been approved.^122^, ^123^, ^124^ One potential avenue to generate a robust tolerance‐inducing response is by directly delivering antigens of interest to the cells of the mucosal immune system. Moreover, a formulation that can deliver tolerance‐inducing molecules, to provide context, along with an antigen can also greatly improve immune responses.

Considerable progress has been made for the development of oral vaccine delivery systems and has also been tested in pre‐clinical models. For example, chitosan and alginate microparticles can be taken up by M cells in the Peyer's patches which can directly be absorbed by the MALT to induce subsequent immune responses.^125^ Polymeric nanoparticles such as poly(lactide‐co‐glycolide) (PLGA) ^126^, ^127^ have also found success in inducing immunoglobulin G (IgG) immune response to promote the linked systemic and mucosal responses necessary for sustained immunity.^128^ Other potential candidates include the encapsulation of antigens or immunomodulatory agents using liposomes,^129^ bilosomes,^130^ bacterial outer membrane vesicles (OMVs),^131^ virus‐like particles (VLPs),^132^ and chemically processed pollen grains,^133^ which have found pre‐clinical success against viral respiratory diseases or bilosome‐entrapped antibiotics with success against the bacterium *Burkholderia pseudomallei*.^130^ The pre‐clinical success of these biomaterials demonstrates how important it is to further our understanding for the enhancement of oral vaccine delivery systems (Table 1).

**TABLE 1:** A representative list of biomaterials as oral drug delivery systems

	Agent type	Examples	Functionality
Mucosa targeting	Polymers	• Thiolated polymers (polycarbophil–cysteine)^51^ • Chitosan‐stearic acid‐thioglycolic acid^52^ • Chitosan^98^, ^99^	• Formation of non‐covalent bonds or stronger covalent bonds to increase residence time • Enhance permeability
	Mucolytic enzymes	• Papain^57^ • Bromelain^57^	• Conjugated on particle surface to cleave mucus substructures • Degraded in gastric environment
	Self‐nanoemulsifying drug delivery systems (SNEDDS)	• Captex 300‐Kollipor EL‐propylenglycol^58^	• Homogenous mixtures of oil, surfactant, and co‐solvent to self‐emulsify in aqueous medium • Ideal for poorly water‐soluble drugs
M‐cell targeting	Plant lectins	• *Ulex europaeus agglutinin* 1 (UEA‐1) lectin^115^	• Possible antinutritional and toxic effects • High affinity to M‐cells as well as glycoproteins
	Proteins	• Protein sigma1^109^ • Claudin 4 targeting peptide (CPE)^113^	• Facilitates oral tolerance in pre‐clinical studies
Epithelia targeting	Transient permeabilizing agents	• Ethylenediaminetetraacetic acid (EDTA)^81^ • Gastrointestinal permeation enhancement technology (GIPET)^83^ • Chiasma's transient permeability enhancer (TPE)^85^	• Enhances transcellular permeation • Less toxicity associated with reversibility
	Surfactants	• Sodium dodecyl sulfate (SDS)^78^ • Polysorbate 80 (PS‐80)^80^	• Amphiphilic structure decreases surface tension • Facilitates epithelial tight junction opening • Irreversible membrane damage
	Bacterial surface protein	• Zonula occludens toxin (Zot)^50^, ^86^	• Rearrangement of epithelial cytoskeleton for tight junction opening • Reversible with no significant toxicity in pre‐clinical studies

## 9 CONCLUSION

Oral routes of administration play a significant role in drug delivery. They prove to be an effective alternative to injected routes of administration due to their high patient compliance and convenience for achieving a specialized immune response. However, even with their copious advantages, numerous orally delivered drugs are associated with low bioavailability. This is generally attributed to degradative conditions and biological barriers, such as the mucosa or epithelia.^134^ Nevertheless, particulate systems that utilize various mucoadhesive and permeabilizing technologies have found success in the clinical translation of these formulated carriers. However, further research is needed for these methods to be utilized for immune engineering. These novel technologies must also be careful to not disrupt or destroy the natural immune function of the GI tract when drugs are delivered orally and therefore should be both transient and effective in its design. Sublingual and buccal routes of administration are also effective methods of immunotherapeutic delivery that differ from the traditional method of oral drug administration. These routes bypass the first‐pass metabolic effect and allow for rapid onset of effects. However, only few developments using these delivery methods have been U.S. FDA approved, due to the uniqueness of its formulations and need for proof of safety and efficacy. Nevertheless, it is important to consider them as viable options for immunotherapeutic delivery. Convenience of sustained administration and high patient compliance make oral routes of administration more attractive methods of immunotherapeutic delivery, as opposed to injectable deliveries. It is expected that the future research in these systems will revolve around immunoengineering concepts for constructing biomaterials that target various cells and organs of the immune system while upholding the integrity of the GI tract as a whole.

## CONFLICT OF INTEREST

There is no conflict of interest.

## AUTHOR CONTRIBUTIONS

**Tien Le:** Conceptualization; writing ‐ original draft; writing‐review & editing. **Brian Aguilar:** Writing ‐ original draft; writing‐review & editing. **Joslyn Mangal:** Conceptualization; writing ‐ original draft; writing‐review & editing.

### PEER REVIEW

The peer review history for this article is available at https://publons.com/publon/10.1002/btm2.10243.

## Data Availability

Any information pertaining to this manuscript will be provided by the authors upon request.
